# Update to an evaluation of ICD-11 PTSD and complex PTSD criteria in a sample of adult survivors of childhood institutional abuse by Knefel & Lueger-Schuster (2013): a latent profile analysis

**DOI:** 10.3402/ejpt.v6.25290

**Published:** 2015-01-02

**Authors:** Matthias Knefel, Donn W. Garvert, Marylene Cloitre, Brigitte Lueger-Schuster

**Affiliations:** 1 Faculty of Psychology, University of Vienna, Vienna, Austria; 2 National Center for PTSD, Veterans Affairs Palo Alto Health Care System, Palo Alto, CA, USA; 3 Department of Psychiatry and Child and Adolescent Psychiatry, Langone Medical Center, New York University, New York, NY, USA

**Keywords:** Complex PTSD, posttraumatic stress disorder, institutional abuse, WHO, ICD-11, latent profile analysis, childhood abuse

## Abstract

**Background:**

The World Health Organization (WHO) International Classification of Diseases, 11th version (ICD-11), has proposed a trauma-related diagnosis of complex posttraumatic stress disorder (CPTSD) separate and distinct from posttraumatic stress disorder (PTSD).

**Objective:**

To determine whether the symptoms endorsed by individuals who had experienced childhood institutional abuse form classes that are consistent with diagnostic criteria for ICD-11 CPTSD as distinct from PTSD.

**Methods:**

A latent profile analysis (LPA) was conducted on 229 adult survivors of institutional abuse using the Brief Symptom Inventory and the PTSD Checklist—Civilian Version to assess current psychopathological symptoms.

**Results:**

The LPA revealed four classes of individuals: (1) a class with elevated symptoms of CPTSD (PTSD symptoms and disturbances in self-organization); (2) a class with elevated symptoms of PTSD and low disturbances in self-organization; (3) a class with elevated disturbances in self-organization symptoms and some elevated PTSD symptoms; and (4) a class with low symptoms.

**Conclusions:**

The results support the existence of a distinct group in our sample, that could be described by the proposed diagnostic category termed CPTSD more precisely than by normal PTSD. In addition, there seems to be a group of persons that do not fulfill the criteria for a trauma-related disorder but yet suffer from psychopathological symptoms.

The World Health Organization (WHO) is in the process of revising the International Classification of Diseases for its 11th edition (ICD-11). Proposals for mental disorders specifically associated with stress have been published by the associated working group (Maercker et al., 2013). The proposals include a new diagnosis termed complex posttraumatic stress disorder (CPTSD) in addition to and distinct from posttraumatic stress disorder (PTSD). The working group defines CPTSD as an extensive reaction typically arising from severe and prolonged stressors such as repeated child sexual abuse, severe domestic violence, torture, or slavery (Maercker et al., 2013). In addition to the PTSD symptoms, CPTSD is comprised of disturbances in the domains of affect, self-concept, and relational functioning (Maercker et al., 2013). The WHO working group process and decision making was guided by the principle of clinical utility and proposed the distinction between PTSD and CPTSD as consistent with this notion (see Cloitre, Garvert, Brewin, Bryant, & Maercker, 2013). Empirical evaluation of the proposed symptom clusters for PTSD and for CPTSD has demonstrated good internal consistency and construct validity (Cloitre et al., 2013; Elklit, Hyland, & Shevlin, 2014; Knefel & Lueger-Schuster, 2013).

The confirmatory factor analysis performed by Knefel and Lueger-Schuster (2013) supported the construct validity of CPTSD. We wished to extend that investigation by conducting latent profile analyses (LPAs) to assess whether the sample participants would fall into distinct classes consistent with the proposed PTSD and CPTSD and thus determine whether the class distinctions found in the above studies (Cloitre et al., 2013; Elklit, Hyland, & Shevlin, 2014) would replicate in this predominantly male sample of adult survivors of childhood institutional abuse. Individuals in the sample experienced maltreatment in institutional settings, defined as an inappropriate use of power and authority, including the potential to harm a child's well-being and development (Wolfe, Jaffe, Jette, & Poisson, 2003) over a longer period of time. Maltreatment included physical, sexual, and emotional abuse (Knefel & Lueger-Schuster, 2013). We expected to identify three different groups of individuals: (1) a group with elevated PTSD symptoms and affect, self-concept, and interpersonal symptoms (disturbances in self-organization), consistent with the CPTSD diagnosis; (2) a group with elevated PTSD symptoms but low level of symptoms related to self-organization, consistent with the ICD-11 PTSD diagnosis; and (3) a group with no elevation in trauma-specific symptoms. We then compared the observed classes regarding differences in severity of symptoms as well as differences in the proportion of individuals meeting the ICD-11 CPTSD and PTSD diagnoses across the classes.

## Method

### Participants and procedure

Participants of this study were the same as in Knefel and Lueger-Schuster (2013). All (*n*=229) were adult survivors of childhood institutional abuse within the Catholic Church and within a federal organization for foster children. The age ranged from 24 to 80 years, the mean age of the sample was 55.8 years (*SD*=9.8), 177 individuals were men (77.3%), and 52 were women (22.7%). The civil status of the sample was representative for the Austrian population, with the majority being married/cohabited (Knefel & Lueger-Schuster, 2013; Lueger-Schuster et al., 2013). All were Caucasians without any migrant background.

All participants experienced at least one type of childhood institutional abuse (physical abuse, sexual abuse, emotional abuse), whereas 86.9% experienced at least two types of violence (see Knefel & Lueger-Schuster, 2013 for details).

### Measures

We used items from two measures to assess psychopathological symptoms: the PTSD Checklist—Civilian Version (PCL-C; Weathers, Litz, Herman, Huska, & Keane, 1991) and the Brief Symptom Inventory (BSI; Derogatis & Melisaratos, 1983). The specific items to assess PTSD and CPTSD symptoms were selected according to the proposed criteria (Cloitre et al., 2013; Maercker et al., 2013), resulting in a total of 12 items, seven from the PCL-C and five from the BSI (see Knefel & Lueger-Schuster, 2013). The PCL-C assesses 17 PTSD symptoms on a five-point Likert scale (1=“none” to 5=“very”). The BSI is a 53-item self-report psychological symptom inventory with nine primary symptom dimensions where symptom distress is similarly rated on a five-point Likert scale (0=“not at all” to 4=“extremely”).

Both measures have proved good psychometric properties (Derogatis & Melisaratos, 1983; Weathers et al., 1991). To compute the ICD-11 diagnoses, which required that the presence of symptoms be categorically defined, we used cut-off points in the PCL-C and BSI that referenced the same symptom level of distress in both questionnaires (2=“moderately” for the PCL-C and 3=“moderately” for the BSI) for the items relevant to the diagnoses.

### Statistical analyses

#### Latent profile analysis

In order to determine the optimal number of classes in this study population, the Lo-Mendell-Rubin adjusted likelihood ratio test (LMR-A), the bootstrap likelihood ratio test (BLRT), and the Bayesian Information Criterion (BIC) were used. In a simulation study, the BLRT was the most consistent indicator of classes, and among the information criteria examined, the BIC performed the best (Nylund, Asparouhov, & Muthen, 2007). The general practice of LPA is to test the fit of a two-class model and systematically increase the number of classes until adding more classes is no longer warranted. The LMR-A and the BLRT compare the fit of the specified class solution to models with one less class. A *p*<0.05 suggests that the specified model provides a better fit to the data relative to the model with one less class. The BIC provides information about model fit with lower relative values indicating improved model fit. A total of 12 raw items were used in the LPA, and gender was included as a covariate. A total of 12 items were used for CPTSD which included six ICD-11 PTSD items and six items for the disturbances in self-organization unique to complex PTSD (see Cloitre et al., 2013; Maercker et al., 2013). The ICD-11 PTSD diagnosis was comprised only of the six ICD-11 PTSD items used in the CPTSD analysis.

## Results

### Latent profile analysis

The two- and three-class models yielded a significant LMR-A and BLRT result at *p*<0.05. The four- and five-class model yielded a significant BLRT result at *p*<0.05, but not a significant LMR-A result at *p*>0.05. A six-class model was examined, but the best log-likelihood value was not replicated and it was not considered for the final model as the *p*-value may not be trustworthy due to local maxima. Although the four-class model did not have a significant LMR-A result, it did have a significant BLRT result and also had the lowest BIC value of all models examined; therefore, it was selected over the other models examined. The fit indices of the different class models can be seen in Table 1.

**Table 1:** Latent class models and fit indices

Model	Log-likelihood	BIC	Entropy	LMR-A *p*	BLRT *p*
2 Classes	−4293.386	8793.253	0.932	<0.0001	<0.0001
3 Classes	−4199.198	8680.950	0.884	0.0135	<0.0001
4 Classes	−4151.242	8661.110	0.901	0.5351	<0.0001
5 Classes	−4116.582	8667.863	0.897	0.7018	<0.0001
6 Classes	−4085.218	8681.207	0.908	0.4714	<0.0001^a^

a The best log-likelihood value was not replicated in 30 out of 50 bootstrap draws. The *p*-value may not be trustworthy due to local maxima.

BIC=Bayesian Information Criterion; LMR-A=Lo-Mendell-Rubin adjusted likelihood ratio test; BLRT=bootstrap likelihood ratio test.

**Fig. 1:**
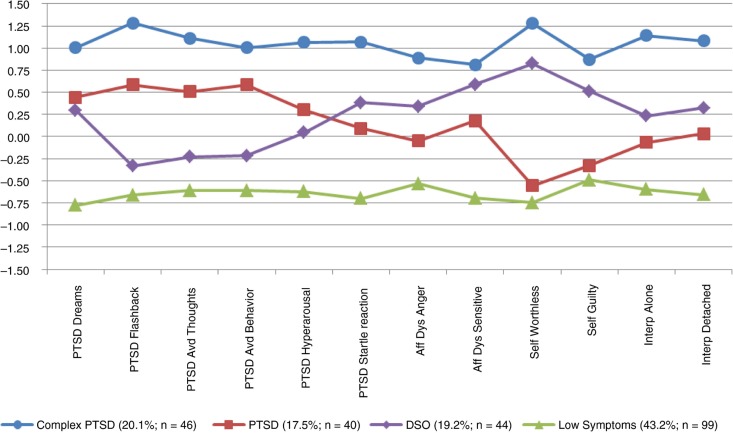
Standardized symptom severity profile by class. For clarity of presentation, the scores depicted in the figure have been standardized.

The four classes were then compared on the 12 items of symptom severity that were used in the LPA in order to provide descriptive labels for each class. Class 1 was labeled as complex PTSD or “CPTSD” as this class had higher levels of symptom severity across the PTSD and disturbances in self-organization when compared to the other classes. Class 2 was labeled as “PTSD” as this class had elevated symptom severity on the PTSD symptoms, but relatively low symptom severity on disturbances in self-organization. Class 3 was labeled as Disturbances in Self-Organization or “DSO” as this class had relatively low symptom severity on the PTSD symptoms but somewhat elevated disturbances in self-organization. Finally, Class 4 was labeled as “Low Symptoms” as this class had relatively low symptom severity on all the symptoms across all domains. The mean standardized values of the items by class are shown in Fig. 1.

The average probability of latent profile membership in the four-class model was acceptable: 0.975 for the CPTSD class, 0.918 for the PTSD class, 0.927 for the DSO class, and 0.958 for the Low Symptoms class, which implies acceptable discrimination between the classes. An acceptable entropy value probability of 0.901 lends support to this result by suggesting adequate class separation. Overall, 20.1% (*n*=46) of participants were classified into the CPTSD class, 17.5% (*n*=40) into the PTSD class, 19.2% (*n*=44) into the DSO class, and 43.2% (*n*=99) into the Low Symptoms class. Interestingly, a gender effect was found between the classes. Females had significantly higher odds of being in the CPTSD class (OR=3.630, *p*<0.001) than males, and females had significantly lower odds of being in the Low Symptoms class (OR=0.399, *p*=0.008) than males. There was not a significant gender effect in the PTSD or in the DSO classes.

### ICD-11 diagnoses by class

We identified the proportion of individuals across the four classes that met the proposed ICD-11 PTSD criteria and the CPTSD criteria. There was a distinct overlap between the diagnoses and the corresponding classes. Most individuals with a PTSD diagnosis were in the PTSD class (59.0%), and most individuals with a CPTSD diagnosis were in the CPTSD class (75.5%). Only three individuals allocated to the Low Symptoms class had PTSD. However, there was a substantial minority of individuals in the DSO class who met either the CPTSD (22.4%) or PTSD (17.9%) diagnosis. We also assessed two types of subthreshold CPTSD (Knefel & Lueger-Schuster, 2013). The proportion of individuals with the subthreshold variants in the CPTSD and PTSD class did not change much. However, 23.6% of subthreshold1 CPTSD and 32.3% of the subthreshold2 CPTSD group fell into the DSO class (Table 2).

**Table 2:** Rates of PTSD, CPTSD, and subthreshold CPTSD in all classes

	Classes				
		
	CPTSD	PTSD	DSO	Low Symptoms	
		
Diagnoses	Class 1	Class 2	Class 3	Class 4	Sign. test
*N* (%) of sample with ICD-11 PTSD (*n*=39)	6 (15.4)	23 (59.0)	7 (17.9)	3 (7.7)	*p*<0.001 2>1, 3, 4
*N* (%) of sample with ICD-11 CPTSD (*n*=49)	37 (75.5)	1 (2.0)	11 (22.4)	0 (0.0)	*p*<0.001 1>2, 3, 4
*N* (%) of sample with ICD-11 sub1CPTSD^a^ (*n*=55)	40 (72.7)	2 (3.6)	13 (23.6)	0 (0.0)	*p*<0.001 1>2, 3, 4
*N* (%) of sample with ICD-11 sub2CPTSD^b^ (*n*=62)	40 (64.5)	2 (3.2)	20 (32.3)	0 (0.0)	*p*<0.001 1>2, 3, 4

a sub1CPTSD consists of at least one re-experiencing and either one avoidance or sense of threat symptom with no changes in the proposed additional symptoms for CPTSD.

b sub2CPTSD consists of at least any two PTSD criteria, with no changes in the proposed additional symptoms for CPTSD.

Symptom level differences across the classes were also assessed. The mean sums of the reported symptoms are given in Table 3 for all four classes. As expected, the Low Symptoms class has the lowest sums in all dimensions. In contrast, individuals in the CPTSD class report the highest values in all dimensions. The DSO class was significantly lower on all symptoms relative to the CPTSD class. However, when compared to the PTSD class, the DSO class was not significantly different in severity of sense of threat symptoms and was significantly higher on self-organization disturbances, specifically affect regulation and negative self-concept.

**Table 3:** Symptom characteristics of the four classes

	CPTSD	PTSD	DSO	Low Symptoms	
		
Characteristics	Class 1	Class 2	Class 3	Class 4	Sign. test
PTSD	18.02 (2.77)	12.58 (2.81)	8.90 (3.55)	3.47 (2.53)	*p*<0.001 1>2>3>4
Re-experiencing	6.09 (1.34)	4.38 (1.58)	2.93 (1.90)	0.91 (1.19)	*p*<0.001 1>2>3>4
Avoidance	6.11 (1.37)	4.70 (1.94)	2.52 (1.74)	1.44 (1.69)	*p*<0.001 1>2>3>4
Sense of threat	5.87 (1.66)	3.50 (1.87)	3.52 (1.98)	1.12 (1.39)	*p*<0.001 1>2, 3>4
Self-organization	17.09 (3.42)	7.78 (2.94)	12.69 (3.04)	3.65 (2.97)	*p*<0.001 1>3>2>4
Affect dysregulation	5.70 (2.03)	3.50 (1.80)	4.58 (2.28)	1.57 (1.57)	*p*<0.001 1>3>2>4
Negative self-concept	5.36 (1.72)	1.28 (1.21)	4.28 (1.26)	0.82 (1.20)	*p*<0.001 1>3>2, 4
Interpersonal problems	6.07 (1.53)	2.95 (1.69)	3.77 (2.01)	1.26 (1.43)	*p*<0.001 1>2, 3>4

## Discussion

In the present paper, we tested whether distinct groups of individuals could be observed, that are characterized by symptom profiles consistent with PTSD and CPTSD as defined by the WHO working group (Maercker et al., 2013) in a sample of adult survivors of childhood institutional abuse. The results from the LPA support the proposed concept of two distinct groups and our hypotheses that three classes would emerge (PTSD, CPTSD, and Low Symptoms). In addition, gender differences were observed where women had significantly higher odds of being in the CPTSD class, and lower odds of being in the Low Symptoms class. The frequently reported gender differences in PTSD rates (e.g., Tolin & Foa, 2006) might be attributable to women's higher likelihood of CPTSD rather than simple PTSD.

It should be noted, however, that a four-class model was the best fit to the data. In addition to the expected three classes, we found a fourth class. Individuals in this class, which we have labeled the “Disturbances in Self-Organization” class, reported elevated symptoms of affect, self-concept, and interpersonal problems as well as having disturbing dreams and being jumpy or easily startled. We have considered two possibilities. First, we considered that the third class might represent individuals with a distinct class of subthreshold CPTSD. However, as presented in Table 2, the proportion of participants with the subthreshold1 CPTSD and subthreshold2 CPTSD were not particularly high in the DSO class (23.6 and 32.6%, respectively), with most individuals who had subthreshold CPTSD still found to fit best as member of the CPTSD class (72.7 and 64.5%, respectively). A second possibility is that this group may be characterized by other axis I and/or axis II disorders. It has been demonstrated that traumatic events during childhood are associated with personality disorders (Bierer et al., 2003), especially with borderline personality disorder (Bandelow et al., 2005; Yen et al., 2002) as well as axis I disorders (Galatzer-Levy, Nickerson, Litz, & Marmar, 2013). In addition, given the specific symptom profile of these individuals (elevated sense of threat and some level of DSO), anxiety and fear disorders might describe them adequately. However, future research is necessary which includes larger and more varied samples and in which axis I and axis II disorders are assessed.

This brief communication reports on the replication of the ICD-11 distinction of groups of individuals best described by PTSD and CPTSD in a fairly specific, predominantly male population who experienced institutional abuse. These finding contribute to the growing evidence of these latent classes in a variety of samples. In addition, investigation of institutional abuse is of growing importance and systematic characterization of individuals with this type of childhood trauma is important. Lastly, we identified a fourth class of individuals and ruled out that they represented variations of PTSD or CPTSD, suggesting an as yet uncharacterized group of individuals who may be suffering from posttraumatic consequences that represent a mix of other disorders.

Given the growing evidence for CPTSD, next steps require a comparison of the extent to which ICD-11 CPTSD patient populations overlap with DSM-5 populations, particularly those with the dissociative subtype. It will also be important to assess the presence of other disorders as well as dimensional differences in symptom burden in future studies.

### Limitations

We did not use CPTSD specific instruments. It is unknown how well the BSI symptom items reflect affect dysregulation and negative self-concept. The ICD-11 diagnoses also require functional impairment, which we did not measure.

## Conclusions

We replicated findings identifying two distinct classes of individuals with symptom profiles consistent with the proposed ICD-11 PTSD and CPTSD diagnoses among men and women who experienced childhood institutional abuse.

## Supplementary Material

Update to an evaluation of ICD-11 PTSD and complex PTSD criteria in a sample of adult survivors of childhood institutional abuse by Knefel & Lueger-Schuster (2013): a latent profile analysis

Update to an evaluation of ICD-11 PTSD and complex PTSD criteria in a sample of adult survivors of childhood institutional abuse by Knefel & Lueger-Schuster (2013): a latent profile analysis

Update to an evaluation of ICD-11 PTSD and complex PTSD criteria in a sample of adult survivors of childhood institutional abuse by Knefel & Lueger-Schuster (2013): a latent profile analysis
