# Supplementary material for: Update to an evaluation of ICD-11 PTSD and complex PTSD criteria in a sample of adult survivors of childhood institutional abuse by Knefel & Lueger-Schuster (2013): a latent profile analysis
Source: Eur J Psychotraumatol. 2015 Jan 2;6:10.3402/ejpt.v6.25290. doi: 10.3402/ejpt.v6.25290 (PMC4283031; doi:10.3402/ejpt.v6.25290)
Supplement: Update to an evaluation of ICD-11 PTSD and complex PTSD criteria in a sample of adult survivors of childhood institutional abuse by Knefel & Lueger-Schuster (2013): a latent profile analysis [file EJPT-6-25290-s001.pdf]

Final but un-edited version

Update zu *Eine Evaluation der ICD-11 PTBS und komplexen PTBS Kriterien anhand einer Stichprobe Erwachsener mit Missbrauchserfahrungen in ihrer Kindheit in Institutionen* von Knefel und Lueger-Schuster (2013) : Eine latente Profilanalyse.

Autoren

Matthias Knefel1*, Donn W. Garvert[2], Marylene Cloitre[2,3], Brigitte Lueger-Schuster[1]

AFFILIATIONS

[1] University of Vienna, Faculty of Psychology, Liebiggasse 5, Vienna, Austria

[2] National Center for PTSD, Veterans Affairs Palo Alto Health Care System, Palo Alto, CA, USA

[3] Department of Psychiatry and Child and Adolescent Psychiatry, New York University, Langone Medical Center, New York, NY, USA

* Korrespondenz: Matthias.Knefel@univie.ac.at

Danksagung
Wir danken Asisa Butollo, Tobias Glück, Reinhold Jagsch, Viktoria Kantor, Yvonne Moy und Dina Weindl für ihre wertvollen Beiträge zu dieser Forschung.

ABSTRACT

Hintergrund: Die Arbeitsgruppe der WHO zur 11. Version der Internationalen Klassifikation psychischer Störungen (ICD-11) hat vorgeschlagen, eine neue Traumafolgestörung, die Diagnose Komplexe Posttraumatische Belastungsstörung (kPTBS) einzuführen. Diese ist von der posttraumatischen Belastungsstörung (PTBS) abzugrenzen und zu unterscheiden.

Ziele: Festzustellen ob die Symptome, die von Individuen berichtete werden, die in ihrer Kindheit Gewalt in Institutionen erlebt haben, Klassen bilden, die mit den diagnostischen Kriterien der ICD-11 für kPTBS übereinstimmen und sich von denen für PTBS unterscheiden.

Methoden: Eine latente Profilanalyse (LPA) wurde mit den Daten 229 erwachsener Überlebender institutioneller Gewalt durchgeführt. Dabei wurde das Brief Symptom Inventory (BSI) und die PTBS Checkliste - für Zivilpersonen (PCL-C) eingesetzt, um aktuelle psychopathologische Symptome zu erheben.

Ergebnisse: Die LPA ergab vier Gruppen von Individuen: (1) eine Gruppe mit verstärkten Symptomen von kPTBS (d.h. PTBS Symptome und Probleme der Selbstregulation); (2) eine Gruppe mit verstärkten Symptomen der PTBS und geringen Problemen der Selbstregulation; (3) eine Gruppe mit erhöhten Problemen der Selbstregulation und ausgewählten PTBS Symptomen; (4) eine Gruppe mit geringer Symptomatik.

Schlussfolgerung: Die Ergebnisse unterstützen die Existenz einer distinkten Gruppe in dieser Stichprobe, die durch die vorgeschlagene diagnostische Kategorie kPTBS besser beschrieben werden kann als durch die einfache PTBS. Zusätzlich scheint es eine Gruppe von Personen zu geben, die die Kriterien für eine Traumafolgestörung nicht erfüllen und trotzdem unter psychopathologischen Symptomen leiden.

Schlüsselwörter: Komplexe PTBS; Posttraumatische Belastungsstörung; institutioneller Missbrauch; WHO; ICD-11; latente Profilanalyse; Missbrauch in der Kindheit;

EINLEITUNG

Die Weltgesundheitsorganisation (WHO) überarbeitet derzeit die *Internationale Klassifikation psychischer Störungen* für ihre elfte Auflage (ICD-11). Die WHO Arbeitsgruppe hat dazu Vorschläge für psychische Störungen, die in Zusammenhang mit traumatischem Stress stehen, publiziert (Maercker et al., 2013). Die Vorschläge beinhalten eine neue Diagnose, genannt Komplexe Posttraumatische Belastungsstörung (kPTBS), zusätzlich zu und abgegrenzt von der Posttraumatischen Belastungsstörung (PTBS).
Die Arbeitsgruppe definiert die kPTBS als eine starke Reaktion, typischerweise auf eine langanhaltende und schwere Stresssituation wie beispielsweise wiederholten sexuellen Missbrauch in der Kindheit, schwere häusliche Gewalt, Folter oder Sklaverei (Maercker et al., 2013). Zusätzlich beinhaltet die Definition von kPTBS Defizite in der Affektregulation, ein negatives Selbstkonzept, interpersonale Probleme und dissoziative Symptome (Maercker et al., 2013). Der Arbeits- und Entscheidungsprozess der WHO-Arbeitsgruppe war geleitet von dem Prinzip der klinischen Nützlichkeit; auf dieser Idee basierend entstand der Vorschlag der neuen Diagnose kPTBS (siehe Cloitre, Gavert, Brewin, Bryant & Maercker, 2013). Die empirische Evaluation der vorgeschlagenen Symptomgruppen für PTBS und kPTBS zeigten eine gute interne Konsistenz und Konstruktvalidität (Cloitre et al., 2013; Elklit, & Shevlin, 2014; Knefel & Lueger-Schuster, 2013).

Die konfirmatorische Faktorenanalyse, durchgeführt von Knefel und Lueger-Schuster (2013), unterstützt die Konstruktvalidität der kPTBS. Die Analyse sollte durch eine Latente Profilanalyse (LPA) erweitert werden, um beurteilen zu können, ob sich voneinander unterscheidbare Gruppen bilden, die den Vorschlägen zu PTBS und kPTBS entsprechen. Weiters zielten wir darauf ab, die bisherigen Forschungsergebnisse zu distinkten Gruppen (Cloitre et al., 2013, Elklit; Hyland und Shelvlin, 2014) in unserer überwiegend männlichen Stichprobe von erwachsenen Überlebenden institutioneller Gewalt zu replizieren.

Personen der Stichprobe haben Gewalt in institutionellem Umfeld erlebt. Institutionelle Gewalt wird definiert als unangebrachte Ausübung von Macht und Autorität, inklusive der Wahrscheinlichkeit das Wohlbefinden und die Entwicklung des Kindes über einen länger andauernden Zeitraum zu beeinträchtigen (Wolfe, Jaffe, Jette, Poisson, 2003). Die Gewalt beinhaltete physischen, sexuellen und emotionalen Missbrauch (Knefel und Lueger-Schuster, 2013). Drei sich voneinander unterscheidende Gruppen von Personen wurden erwartet: (1) eine Gruppe mit PTBS Symptomen und Symptomen der Affektregulation, ein negatives Selbstkonzept und interpersonale Probleme (Probleme der Selbstregulation), konsistent mit der kPTBS Diagnose, (2) eine Gruppe mit PTBS Symptomen aber schwächer ausgeprägten Symptomen im Zusammenhang mit der Selbstregulation, konsistent mit der ICD-11 PTBS Diagnose und (3) eine Gruppe mit keinen traumaspezifischen Symptomen. Die beobachteten Gruppen werden hinsichtlich des Schweregrades der gezeigten Symptome unterschieden. Außerdem wurde das Verhältnis der Personen, die den Kriterien der ICD-11 Diagnosen für kPTBS und PTBS entsprechen, über die Gruppen hinweg, verglichen.

METHODIK

Stichprobe und Ablauf

Es handelt sich um dieselbe Stichprobe wie bei Knefel und Lueger-Schuster (2013). Alle (N = 229) waren erwachsene Überlebende institutioneller Gewalt innerhalb der katholischen Kirche und staatlichen Einrichtungen für Pflegekinder. Das durchschnittliche Alter in der Stichprobe lag bei 55.8 Jahren; der jüngste Teilnehmer war 24 Jahre alt und der älteste 80 Jahre alt. Insgesamt wurden Daten von 177 Männern (77.3%) und 52 Frauen (22.7%) analysiert. Der Familienstand der Teilnehmer war repräsentativ für die österreichische Gesamtbevölkerung, wobei die Mehrheit verheiratet war oder in Partnerschaft lebte (Knefel und Lueger-Schuster, 2013; Lueger-Schuster et al., 2013).

Alle Teilnehmer erfuhren zumindest eine Art von Institutioneller Gewalt (physischer, emotionaler oder sexueller Missbrauch). 86,9% haben mindestens zwei Formen der Gewalt erfahren (siehe Knefel und Lueger-Schuster, 2013, für Details).

Instrumente

Es wurden Items von zwei psychologischen Instrumenten herangezogen, um psychopathologische Symptome zu bewerten: die Posttraumatic Stress Disorder Checklist- Civilian Version (PCL-C; Weathers, Litz, Herman, Huska und Keane, 1991) und das Brief Symptom Inventory (BSI; Derogatis und Melisaratos, 1983). Die spezifischen Items, um PTBS und kTPBS Symptome zu erfassen, wurden nach den vorgeschlagenen Kriterien ausgewählt (Cloitre et al., 2013 , Maercker et al., 2013), wodurch sich insgesamt zwölf Items ergaben, sieben davon aus dem PCL-C und fünf aus dem BSI (siehe Knefel und Lueger-Schuster, 2013). Die PCL-C erhebt 17 PTBS Symptome basierend auf dem DSM-IV (APA, 2000). Auf einer fünf-stufigen Likert Skala (1 = „gar nicht" bis 5 = „äußerst") bewerten die Teilnehmer die Symptome, die sie in den vergangen vier Wochen erlebt haben.

Das BSI ist ein Instrument zur Selbsteinschätzung klinisch relevanter psychologischer Symptome mit guter Reliabilität und Validität (Derogatis & Melisaratos, 1983; Franke & Derogatis, 2000). Die Teilnehmer bewerten 53 Items in Bezug auf ihr symptomatisches Leiden auf einer fünf-stufigen Likert Skala (0 = „überhaupt nicht" bis 4 = „sehr stark"). Die 53 Items umfassen neun Symptomdimensionen. Beide Instrumente haben gute psychometrische Eigenschaften bewiesen (Weathers et al., 1991; Derogatis und Melisaratos, 1983). Um die ICD-11 Diagnose zu generieren, wurden Cut-off-Werte für die diagnoserelevanten Items verwendet, die sich in beiden Fragebögen auf dasselbe Symptombelastungslevel beziehen.

Statistische Analysen

Latente Profilanalyse. Um die optimale Anzahl an Klassen in dieser Stichprobe festzustellen wurde der adjusted Lo-Mendel-Rubin Likelihood-Quotiententest Test (LMR-A), der Bootstrap Likelihood Ratio Test (BLRT) und das Bayesian Information Criterion (BIC) verwendet. In einer Simulationsstudie erwies sich der BLRT als am konsistentesten beim Ermitteln von Klassen. Bei den Informationskriterien, die beobachtet wurden, zeigte der BIC die beste Leistung (Nylund et al., 2007). In der gängien Praxis der latenten Profilanalyse wird ein Zwei-Klassen-Modell getestet und dann die Klassenanzahl so lange erhöht, bis kein Informationsgewinn durch Hinzufügen einer weiteren Klasse erreicht wird. Der LMR-A und der BLRT vergleichen das errechnete Klassen-Modell mit einem Modell, das eine Klasse weniger beinhaltet. Ein *p*-Wert $<.05$ weist darauf hin, dass das definierte Modell eine bessere Passung zeigt als

jenes mit einer Klasse weniger. Das BIC stellt Informationen zur Modellpassung zur Verfügung, wobei geringere relative Werte eine Verbesserung der Modellpassung anzeigen. Insgesamt wurden 12 Item-Rohwerte in der LPA verwendet. Geschlecht wurde als Kovariate mit hineingenommen. 12 Items wurden verwendet, um kPTBS zu erheben. Diese 12 beinhalteten sechs ICD-11 PTBS Items und sechs Items, um die Störungen in der Selbstregulation zu erheben (siehe Cloitre et al., 2013; Maercker et al., 2013). Die ICD-11 PTBS Diagnose bestand nur aus den sechs ICD-11 PTBS Items, die auch zur Erfassung der kPTBS verwendet wurden.

ERGEBNISSE

Latente Profilanalyse

Die Zwei- und Drei-Klassen-Modelle ergaben signifikante LMR-A und BLRT Werte, $p<.05$. Das Vier- und das Fünf-Klassen Modell ergab signifikante BLRT Werte, $p< .05$, aber keinen signifikanten LMR-A Wert, $p>.05$. Ein sechs Klassen Modell wurde untersucht, aber die beste Log-likelihood Funktion konnte nicht repliziert werden und wurde deswegen nicht als finale Version herangezogen; der *p*-Wert ist aufgrund lokaler Maxima möglicherweise nicht zulässig. Obwohl das Vier-Klassen Modell keine signifikanten LMR-A Werte hatte, zeigten sich signifikante Werte beim BLRT und es hatte außerdem den niedrigsten BIC Wert von allen untersuchten Modellen; deswegen wurde es den anderen Modellen vorgezogen. Die Fit Indices können in Tabelle 1 nachgelesen werden.

Die vier Klassen wurden dann mit den 12 standardisierten Items zum Symptomschwergrad, die im LPA verwendet wurden, verglichen, um die Klassen benennen zu können. Klasse 1 wurde Komplexe PTBS oder "kPTBS" genannt, weil diese Klasse, im Vergleich zu den anderen Klassen, sowohl die schwersten PTBS Symptome als auch die schwersten Probleme der Selbstregulation zeigten. Klasse 2 wurde "PTBS" genannt, weil sie erhöhte PTBS Symptome zeigte aber relativ geringe Probleme der Selbstregulation. Klasse 3 wurde „Probleme der Selbstregulation" (Disturbances in Self-Organization [DSO]) bezeichnet, da sie eher geringe PTBS Werte zeigten aber erhöhte Selbstregulationsprobleme. Klasse 4 wurde mit "geringe Symptome" beschrieben, weil sie nur relativ geringe Symptome in allen Bereichen zeigten. Die standardisierten Durchschnittswerte können in Abbildung 1 abgelesen werden (Zur besseren Anschaulichkeit werden in Abb.1 standardisierte Werte dargestellt).

Die durchschnittliche Wahrscheinlichkeit einer latenten Klassenzugehörigkeit in den vier Gruppen war akzeptabel: .975 für die kPTBS Gruppe, .918 für die PTBS Gruppe, .927 für die Selbstregulationsproblemgruppe und .958 für die Gruppe mit geringen Symptomen. Dies erlaubt eine akzeptable Unterscheidung zwischen den Klassen. Ein akzeptablerEntropy Wert von .901 spricht auch für diese Klassenlösung. Insgesamt wurden 20,1% (n=46) der Teilnehmer der kPTBS Klasse zugeordnet, 17,5% (n=40) der PTBS Klasse, 19,2% (n=44) der Klasse mit Selbstregulationsproblemen und 43,2% (n=99) der Klasse mit geringen Symptomen. Interessanterweise wurde ein Gendereffekt zwischen den Klassen gefunden. Frauen hatten eine signifikant höhere Wahrscheinlichkeit der kPTBS Klasse zugeordnet zu werden (OR=3.630, p kleiner als .001) als Männer. Frauen wurden außerdem signifikant weniger häufig der Gruppe mit geringen Symptomen zugeordnet (OR=0.399, p=.008) als Männer. Es zeigte sich kein signifikanter Gendereffekt in der PTBS oder der Klasse mit Selbstregulationsproblemen.

Tabelle 1. Latente Klassen Modelle und Fit Indices.

Formaterat: Tyska (Österrike)

Modell	Log-likelihood	BIC	Entropy	LMR-A *p*-Wert	BLRT *p*-Wert
2 Klassen	-4293.386	8793.253	.932	<.0001	<.0001
3 Klassen	-4199.198	8680.950	.884	.0135	<.0001
4 Klassen	-4151.242	8661.110	.901	.5351	<.0001
5 Klassen	-4116.582	8667.863	.897	.7018	<.0001
6 Klassen	-4085.218	8681.207	.908	.4714	<.0001*

Anmerkung. BIC, Bayesian information criterion; LMR-A, Lo-Mendell-Rubin adjusted likelihood ratio test; BLRT, bootstrap likelihood ratio test.
* der beste log-likelihood Wert wurde in 30 von 50 bootstrap Ziehungen nicht repliziert. Der *p*-Wert ist aufgrund lokaler Maxima möglicherweise nicht zulässig.

Abbildung 1. Standardisierte Symptomprofile pro Klasse.

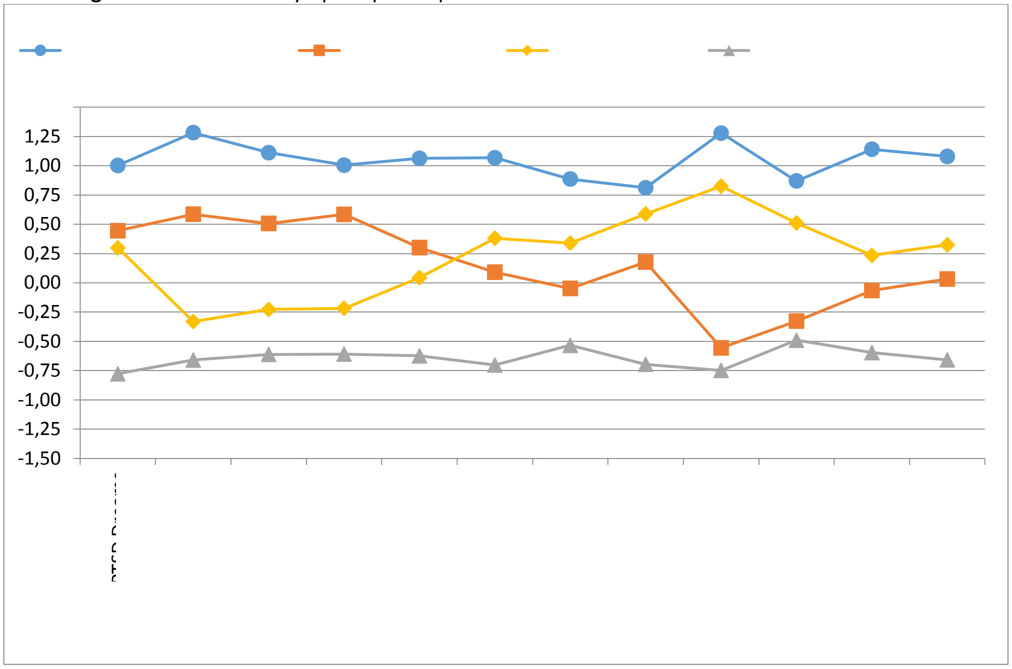

ICD-11 Diagnosen nach Klassen

Es wurde die Verteilung der Personen, die nach den ICD-11 die Kriterien PTBS und kPTBS erfüllten, über die Gruppen, analysiert. Es zeigte sich eine eindeutige Überlappung der Diagnosen mit den entsprechenden Klassen. Die meisten Personen mit einer PTBS Diagnose waren der PTBS Klasse zugeordnet (59,0%) und die meisten Personen mit kPTBS wurden der kPTBS Klasse zugeordnet (75,5%). Nur drei Personen, die der Klasse mit geringen Symptomen zugeordnet wurden, hatten PTBS. Allerdings zeigte sich eine beachtliche Minderheit an Personen, die der Klasse mit Selbstregulationsproblemen

zugeordnet waren aber entweder mit kPTBS (22,4%) oder PTBS (17,9%) diagnostiziert wurden. Außerdem wurden zwei Gruppen mit subklinischer kPTBS diagnostiziert (Knefel und Lueger-Schuster, 2013). Unter Berücksichtigung dieser subklinischen Diagnosen änderte sich nur geringfügig etwas an der Verteilung. Allerdings fielen 23,6% der subklinischen1 kPTBS Gruppe und 32,3% der subklinischen2 kPTBS Gruppe unter die Gruppe mit Selbstregulationsproblemen (Tabelle 2).

Tabelle 2.

Diagnosen	Klassen				Sign. Test
	kPTBS Klasse 1	PTBS Klasse 2	DSO Klasse 3	geringe Symptome Klasse 4	
N (%) der TN mit ICD-11 PTBS (n=39)	6 (15.4)	23 (59.0)	7 (17.9)	3 (7.7)	*p*<.001 2>1,3,4
N (%) der TN mit ICD-11 kPTBS (n=49)	37 (75.5)	1 (2.0)	11 (22.4)	0 (0.0)	*p*<.001 1>2,3,4
N (%) der TN mit ICD-11 sub1kPTBS[a] (n=55)	40 (72.7)	2 (3.6)	13 (23.6)	0 (0.0)	*p*<.001 1>2,3,4
N (%) der TN mit ICD-11 sub2kPTBS[b] (n=62)	40 (64.5)	2 (3.2)	20 (32.3)	0 (0.0)	*p*<.001 1>2,3,4

[a]sub1kPTBS, zumindest ein Wiedererleben-Symptom und entweder ein Vermeidungssymptom oder ein Gefühl in Gefahr zu sein Symptom und keine Änderung in den kPTBS Symptomen. [b]sub2kPTBS, zumindest irgendwelche zwei PTBS Kriterien werden erfüllt und keine Änderung in den kPTBS Symptomen.

Unterschiede in der Symptombelastung zwischen den Klassen wurden analysiert und die Durchschnittswerte pro Klasse sind in Tabelle 3 dargestellt. Wie zu erwarten, zeigte die Klasse mit geringen Symptomen die geringsten Durchschnittswerte in allen Dimensionen. Im Gegensatz dazu berichteten die Personen der kPTBS Klasse die höchsten Werte in allen Dimensionen. Die Selbstregulationsproblem-Klasse zeigte weniger Symptome im Vergleich zur kPTBS Klasse. Allerdings zeigte die Klasse mit Selbstregulationsproblemen, im Vergleich zur PTBS Klasse, keinen höheren Werte im Gefühl in Gefahr zu sein aber zeigte höhere Werte in den Probleme der Selbstregulation, speziell in der Affekt-Dysregulation und im negativen Selbstkonzept.

Tabelle 3. Symptomcharakteristiken der vier Klassen

Charakteristiken	kPTBS Class 1	PTBS Class 2	DSO Class 3	Geringe Symptome Class 4	Sign. Test
PTBS	18.02 (2.77)	12.58 (2.81)	8.90 (3.55)	3.47 (2.53)	*p*<0.001 1>2>3>4
Wiedererleben	6.09 (1.34)	4.38 (1.58)	2.93 (1.90)	0.91 (1.19)	*p*<0.001 1>2>3>4
Vermeidung	6.11 (1.37)	4.70 (1.94)	2.52 (1.74)	1.44 (1.69)	*p*<0.001 1>2>3>4
Gefühl in Gefahr zu sein	5.87 (1.66)	3.50 (1.87)	3.52 (1.98)	1.12 (1.39)	*p*<0.001 1>2,3>4

Selbst-regulation	17.09 (3.42)	7.78 (2.94)	12.69 (3.04)	3.65 (2.97)	*p*<0.001 1>3>2>4
Affekt-Dysregulation	5.70 (2.03)	3.50 (1.80)	4.58 (2.28)	1.57 (1.57)	*p*<0.001 1>3>2>4
Negatives Selbstkonzept	5.36 (1.72)	1.28 (1.21)	4.28 (1.26)	0.82 (1.20)	*p*<0.001 1>3>2,4
Interpersonale Probleme	6.07 (1.53)	2.95 (1.69)	3.77 (2.01)	1.26 (1.43)	*p*<0.001 1>2,3>4

DISKUSSION

In dem vorliegenden Artikel wurde untersucht, ob voneinander unterscheidbare Gruppen, anhand von Symptomprofilen beobachtet werden können, die sich mit den kPTBS und PTBS Profilen, wie sie von der WHO Arbeitsgruppe beschrieben wurden (Maecker et al., 2013) decken. Die Stichprobe setzte sich aus erwachsenen Überlebenden von institutioneller Gewalt in der Kindheit zusammen. Die Ergebnisse der LPA unterstützen das vorgeschlagene Konzept zwei differenzierbarer Gruppen und die angenommene Hypothese, dass sich drei Klassen zeigen würden (PTBS, kPTBS, geringe Symptome). Zusätzlich wurden Genderunterschiede beobachtet: Frauen hatten signifikant höhere Wahrscheinlichkeit der kPTBS Klasse zugeordnet zu werden und geringere Wahrscheinlichkeit der Klasse mit geringen Symptomen zugeteilt zu werden. Die regelmäßig berichteten Genderunterschiede in PTBS (z.B. Tolin und Foa, 2006) könnten auf die höhere Wahrscheinlichkeit von kPTBS bei Frauen zurückzuführen sein.

Allerdings stellte das Vier-Klassen Modell die Daten am besten dar. Zusätzlich zu den drei erwarteten Klassen, wurde eine vierte Klasse gefunden. Personen in dieser Klasse (Probleme der Selbstregulation), berichteten von erhöhten Werten in der Affektdysregulation, im Selbstkonzept und bei interpersonalen Problemen, sowie auch bei verstörenden Träumen und dem Gefühl der Unruhe und erhöhter Schreckhaftigkeit. Zwei Erklärungen wurden hierzu in Betracht gezogen: Zuerst wurde angenommen, dass die dritte Klasse Personen mit subklinischen kPTBS beinhalte. Allerdings zeigte sich, wie in Tabelle 2 dargestellt, dass der Anteil an Personen mit sub1kPTBS und sub2kPTBS in der Klasse mit Selbstregulationsproblemen nicht besonders hoch war (23,6% bzw. 32,6%). Die meisten Personen mit subklinischer kPTBS passten am besten in die kPTBS Klasse (72,7% bzw. 64,5%). Die zweite Möglichkeit besteht darin, dass diese Gruppe eher an anderen Achse I und/oder Achse II Störungen leidet. Es konnte gezeigt werden, dass traumatische Erlebnisse in der Kindheit mit Persönlichkeitsstörungen assoziiert sind (Bierer et al., 2003), speziell mit Borderline Persönlichkeitsstörung (Bandelow et al., 2005; Yen et al., 2002) und Achse I Störungen (Galatzer-Levy, Nickerson, Litz, Marmar, 2013). Es wäre aufgrund der speziellen Symptomprofile dieser Personen (gesteigertes Gefühl in Gefahr zu sein und einige Symptome der Selbstregulationsproblematik), denkbar, dass sie an Angst- und Furchtstörungen leiden. Weitere Forschung mit größeren Stichproben und unterschiedlichen Gruppen ist notwendig. Außerdem sollten Achse I und Achse II Störungen mit erhoben werden.

Dieser kurze Überblick berichtet über die Replikation der ICD-11 Unterscheidung von Gruppen von Personen, die am besten durch PTBS und kPTBS beschrieben werden. Die Stichprobe war eine sehr spezifische, überwiegend männliche, mit institutioneller Gewalterfahrung in der Kindheit. Diese

Ergebnisse tragen zu den wachsenden Beweisen dieser voneinander unterscheidbaren Gruppen bei. Zusätzlich ist die Untersuchung von institutioneller Gewalt von immer größerer Wichtigkeit. Die systematische Charakterisierung von Personen mit dieser Art von Kindheitstrauma ist wichtig. Interessanterweise wurde eine vierte Klasse identifiziert, wobei es ausgeschlossen wurde, dass es sich hierbei um eine Variation der PTBS oder kPTBS handelt. Möglicherweise handelt es sich bei dieser hier nicht klar beschreibbaren Gruppe um Personen, die an anderen als Trauma-spezifischen Störungen leiden.

Neben der wachsende Evidenz für ICD-11 kPTBS, scheint ein Vergleich der ICD-11 Diagnosen mit den DSM-5 Diagnosen sinnvoll, insbesondere unter Berücksichtigung der kPTBS und des dissoziativen Subtyps von PTBS in DSM-5. In zukünftigen Studien wird es wichtig sein andere Störungen mitzuerheben, sowie auch dimensionale Unterschiede der Symptombelastung zu beurteilen.

Einschränkungen

Es wurden keine speziellen kPTBS Instrumente verwendet. Es ist unbekannt wie gut die BSI-Symptomitems die affektive Dysregulation und das negative Selbstkonzept erfassen. Die ICD-11 Diagnosen erfordern auch funktionelle Beeinträchtigung, welche hier nicht erhoben wurden.

Schlussfolgerungen

Es konnten, in dieser Stichprobe (bestehend aus Männern und Frauen, die institutionelle Gewalt in der Kindheit erlebt haben), zwei anhand von Symptomprofilen voneinander differenzierbare Klassen von Personen identifiziert werden, die konsistent mit den vorgeschlagenen ICD-11 PTBS und kPTBS Diagnosen sind.

Literatur

Bandelow, B., Krause, J., Wedekind, D., Broocks, A., Hajak, G., & Rüther, E. (2005). Early traumatic life events, parental attitudes, family history, and birth risk factors in patients with borderline personality disorder and healthy controls. *Psychiatry Research, 134,* 169-179.

Bierer, L. M., Yehuda, R., Schmeidler, J., Mitropoulou, V., New, A. S., Silverman, J. M., & Siever, L. J. (2003). Abuse and neglect in childhood: relationship to personality disorder diagnoses. *CNS spectrums, 8*, 737-740.

Cloitre, M., Garvert, D. W., Brewin, C. R., Bryant, R. A., & Maercker, A. (2013). Evidence for proposed ICD-11 PTSD and complex PTSD: A latent profile analysis. *European Journal of Psychotraumatology*, *4*. doi: 10.3402/ejpt.v4i0.20706.

Derogatis, L. R., & Melisaratos, N. (1983). *The brief symptom inventory: An introductory report*. Psychological Medicine*, 3*, 595-605.

Tolin, D. F., & Foa, E. B. (2006). Sex differences in trauma and posttraumatic stress disorder: A quantitative review of 25 years of research. *Psychological Bulletin, 132*, 959-992.

Elklit, A., Hyland, P., & Shevlin, M. (2014). Evidence of symptom profiles consistent with posttraumatic stress disorder and complex posttraumatic stress disorder in different trauma samples. *European Journal of Psychotraumatology, 5*. doi:10.3402/ejpt.v5.24221

Galatzer-Levy, I. R., Nickerson, A., Litz, B. T., & Marmar, C. R. (2013). Patterns of lifetime PTSD comorbidity: a latent class analysis. *Depression and Anxiety, 30*, 489-496.

Knefel, M., & Lueger-Schuster, B. (2013). An evaluation of ICD-11 PTSD and complex PTSD criteria in a sample of adult survivors of childhood institutional abuse. European Journal of Psychotraumatology, *4*. doi: 10.3402/ejpt.v4i0.22608

Lueger-Schuster, B., Kantor, V., Weindl, D., Knefel, M., Moy, Y., Butollo, A., . . . Glück, T. (2013). Institutional abuse of children in the Austrian Catholic Church: Types of abuse and impact on adult survivors' current mental health. *Child Abuse & Neglect, 38*, 52–64. doi:10.1016/j.chiabu.2013.07.013

Maercker, A., Brewin, C. R., Bryant, R. A., Cloitre, M., Ommeren, M., Jones, L. M., ... Reed, G. M. (2013). Diagnosis and classification of disorders specifically associated with stress: proposals for ICD -11. *World Psychiatry, 12*, 198-206.

Nylund, K. L., Asparouhov, T., & Muthen, B. O. (2007). Deciding on the number of classes in latent class analysis and growth mixture modeling: A monte carlo simulation study. *Structural Equation Modeling, 14*, 535-569.

Weathers, F. W., Litz, B. T., Herman, D., Huska, J., & Keane, T. M. (1991). *The PTSD Checklist_Civilian Version (PCL-C*). Boston, MA: FW Weathers National Center for PTSD, Boston Veterans Affairs Medical Center.

Wolfe, D. A., Jaffe, P. G., Jette, J. L., & Poisson, S. E. (2003). The impact of child abuse in community institutions and organizations: Advancing professional and scientific understanding. *Clinical Psychology-Science and Practice, 10,* 179–191. dx.doi.org/10.1093/clipsy/bpg021

Yen, S., Shea, M. T., Battle, C. L., Johnson, D. M., Zlotnick, C., Dolan-Sewell, R., ... McGlashan, T. H. (2002). Traumatic exposure and posttraumatic stress disorder in borderline, schizotypal, avoidant, and obsessive-compulsive personality disorders: findings from the collaborative longitudinal personality disorders study. *Journal of Nervous and Mental Disease, 190*, 510-518.

Formaterat: Tyska (Österrike)
