# Supplementary material for: Update to an evaluation of ICD-11 PTSD and complex PTSD criteria in a sample of adult survivors of childhood institutional abuse by Knefel & Lueger-Schuster (2013): a latent profile analysis
Source: Eur J Psychotraumatol. 2015 Jan 2;6:10.3402/ejpt.v6.25290. doi: 10.3402/ejpt.v6.25290 (PMC4283031; doi:10.3402/ejpt.v6.25290)
Supplement: Update to an evaluation of ICD-11 PTSD and complex PTSD criteria in a sample of adult survivors of childhood institutional abuse by Knefel & Lueger-Schuster (2013): a latent profile analysis [file EJPT-6-25290-s002.pdf]

La Rumination Agit-elle sur la Relation entre les Capacités de Régulation Émotionnelle et l'État de Stress Post-Traumatique ?

Thomas Ehring, Anke Ehlers

Abstract
Contexte et objectifs. La rumination liée à un traumatisme a été supposée comme pouvant entretenir l'état de stress post-traumatique (ESPT). Ce point de vue a été consolidé empiriquement par des preuves grandissantes, basées sur des protocoles transversaux, prospectifs et expérimentaux. Cependant, il n'est pas clair pourquoi les survivants d'un traumatisme s'engagent alors dans la rumination en dépit de ses conséquences négatives. La présente étude a pour but d'explorer l'hypothèse selon laquelle de faibles capacités de régulation émotionnelle sous-tendent la rumination liée à un traumatisme.
Méthode. Les capacités de régulation émotionnelle et la rumination liée à un traumatisme ont été évaluées chez N = 93 sujets en situation post-traumatique, 2 semaines après avoir survécu à un accident de la route. De plus, les niveaux symptomatologiques de l'ESPT ont été évalués à 2 semaines, 1 mois, 3 mois et 6 mois de suivi.
Résultats. Les capacités de régulation émotionnelle étaient significativement liées à la rumination traumatique ainsi qu'aux niveaux symptomatologiques de l'ESPT. De plus, la rumination a joué un rôle dans l'association entre de faibles capacités de régulation émotionnelle et l'ESPT.
Conclusions. Ces résultats supportent le fait que la rumination soit utilisée comme stratégie dysfonctionnelle de régulation émotionnelle par les survivants d'un traumatisme.

Keywords: Traumatisme ; ESPT ; Rumination ; Régulation Émotionnelle

Name of translator: Marc Legrand

Citation: European Journal of Psychotraumatology 2014, **5**: 23547 - http://dx.doi.org/10.3402/ejpt.v5.23547
