# Supplementary material for: Update to an evaluation of ICD-11 PTSD and complex PTSD criteria in a sample of adult survivors of childhood institutional abuse by Knefel & Lueger-Schuster (2013): a latent profile analysis
Source: Eur J Psychotraumatol. 2015 Jan 2;6:10.3402/ejpt.v6.25290. doi: 10.3402/ejpt.v6.25290 (PMC4283031; doi:10.3402/ejpt.v6.25290)
Supplement: Update to an evaluation of ICD-11 PTSD and complex PTSD criteria in a sample of adult survivors of childhood institutional abuse by Knefel & Lueger-Schuster (2013): a latent profile analysis [file EJPT-6-25290-s003.pdf]

A BNO-11-es komplex PTSD diagnózis újraértékelése intézményes gyermekkori abúzus felnőtt túlélői körében: egy látens profilelemzés (Knefel & Lueger-Schuster, 2013)

Matthias Knefel, Donn W Garvert, Marylène Cloitre, Brigitte Lueger-Schuster

Abstract
Háttér: A WHO Betegségek Nemzetközi Osztályozása 11. kiadása (BNO-11) egy új, traumával kapcsolatos diagnózist vezetett be, a komplex poszttraumás stressz zavart (CPTSD), amely különbözik és elkülönül a poszttraumás stressz zavar (PTSD) diagnózistól.
Célkitűzés: Annak meghatározása, hogy intézményes abúzuson átesett, a CPTSD-vel konzisztens tünetek mutató személyek tünetei elkülönülnek a PTSD-től.
Módszer: Látens profilelemzést végeztek (LPA) 229 intézményes abúzust túlélő felnőtt személyen a Brief Symptom Inventory-t (BSI) és a PTSD Checklist – Civilian Version-t (PCL-C) felhasználva.
Eredmények: Az LPA alapján négy osztály különült el: (1) emelkedett CPTSD tünetek (PTSD tünetek és az énszerveződés zavara); (2) emelkedett PTSD tünetek, de nincs énszerveződési zavar; (3) énszerveződés zavara, de alacsonyabb szintű PTSD tünetek, (4) nincs tünet.
Következtetések: Az eredmények alátámasztják azt, hogy létezik egy elkülönült csoport, amelynek a tüneteit a CPTSD sokkal pontosabban leírja, mint a PTSD. Továbbá, van egy olyan csoport, amely nem éri el egyik traumával kapcsolatos diagnózis kritériumát sem, de mégis vannak pszichés tünetei.

Keywords: komplex PTSD, poszttraumás stressz zavar, intézményes abúzus, WHO, BNO-11, látens profilelemzés, gyermekkori bántalmazás

Name of translator: Kinga Fodor

European Journal of Psychotraumatology 2014, 5: 25290 - http://dx.doi.org/10.3402/ejpt.v5.25290
